# Fast Tracks and Roadblocks for Zika Vaccines

**DOI:** 10.3390/vaccines6040077

**Published:** 2018-11-21

**Authors:** Khairunnisa Abdul Ghaffar, Lisa F.P. Ng, Laurent Renia

**Affiliations:** 1 Singapore Immunology Network, Agency for Science, Technology and Research (A*STAR), Singapore 138648, Singapore; khairunnisa_ghaffar@immunol.a-star.edu.sg (K.A.G.); lisa_ng@immunol.a-star.edu.sg (L.F.P.N.); 2 Institute of Infection and Global Health, University of Liverpool, Liverpool L69 7BE, UK

**Keywords:** Zika virus, vaccine, immune responses, delivery systems

## Abstract

In early 2014, a relatively obscure virus, the Zika virus, made headlines worldwide following an increase in the number of congenital malformations. Since then, research on Zika virus, treatment and vaccines have progressed swiftly with various drugs being repurposed and vaccines heading into clinical trials. Nonetheless, the need for a vaccine is crucial in order to eradicate this re-emerging arthropod-borne virus which remained silent since its first discovery in 1947. In this review, we focused on how the inconspicuous virus managed to spread, the key immunological factors required for a vaccine and the various vaccine platforms that are currently being studied.

## 1. History of Zika Virus

Zika virus (ZIKV) was first isolated within the Zika forest in Uganda back in 1947 when a group of scientists working on yellow fever unexpectedly discovered an unknown new virus from a sentinel rhesus macaque [1]. It was then characterized and classified as an arthropod-borne virus belonging to the *Flaviviridae* family and *Flavivirus* genus. Years later, the first confirmed human ZIKV infection case came in 1952 when live virus was isolated from a patient in Eastern Nigeria [2]. Since then, it was confirmed that ZIKV is a human viral pathogen. 

The first recorded human outbreak of ZIKV originated on the Island of Yap in 2007 whereby an estimated 73% of the population were infected. Prior to this, there were only 14 reported human ZIKV infections [3]. Subsequently, in 2008, Foy et al., found evidence that human-to-human transmission of ZIKV was plausible through sexual intercourse [4]. Unfortunately, the start of 2012 ushered in a wave of various mosquito-borne diseases which included Zika, Chikungunya and Dengue virus in the Pacific islands [5,6]. A routine check of blood donations in French Polynesia found samples which were positive for ZIKV when tested using PCR, triggering a closer surveillance of the spread of ZIKV [7]. In November 2013, the first suspected case of perinatal transmission of ZIKV was reported in the French Polynesia where a newborn had symptoms of mild pruritic rash at the time of delivery with the mother displaying symptoms of ZIKV infection two weeks prior delivery [8].

Following the recent surge of ZIKV cases in 2013 and 2014, an increase in congenital cerebral malformations, brainstem dysfunction and microcephaly among fetuses and newborns were observed, however the cause was unknown [9]. It was only when the Brazilian Government reported a correlation between ZIKV virus infections with congenital malformations in November 2015 that triggered health authorities to keep a vigilant watch for this inconspicuous disease [10]. By February 2016, a public health emergency was declared following proof of the association between ZIKV, microcephaly and other neurologic disorders such as the Guillain-Barre syndrome.

## 2. Treatment

Due to the massive outbreak of ZIKV infection in 2015 without a known cure, various groups have been working on repurposing FDA-approved drugs for treatment [11,12,13]. Repurposing drugs would save a lot of time spent on finding and testing new molecules as human pharmacological data could be found in the pharmacopoeia; however more studies are needed to address issues such as the concentration of drugs needed to kill 90% of the virus and the drug exposure levels in the relevant organs or tissues. There are currently no FDA approved drugs under investigation in clinical trials solely for the treatment of ZIKV infection [14]. Hence despite efforts to repurpose known drugs for treatment, there is still an urgent need for a vaccine against ZIKV due to its implications for a pregnant mother and child. 

## 3. Challenges for a Zika Vaccine

There are many challenges when designing a vaccine targeting ZIKV. Firstly, there is currently no perfect small animal model for pre-clinical testing. Wild-type (WT) mice are poorly susceptible to Zika infection and develop low and short-lived viremia without apparent pathologies [15,16,17,18]. This is due to the strong type I interferon response during the first days of infection [19,20]. Mice deficient in molecules of this pathway have been shown to sustain high viremia and display a large array of pathologies [16]. WT models includes the use of antibodies against the interferon alpha/beta receptor have also been used extensively as a model [16]. Thus, so far, non-human primates (NHP) which is one of the natural reservoirs of the virus are the best pre-clinical models as discussed in depth by Lee & Ng [19]. The cost and maintenance of testing vaccines on NHP is extremely high and this poses a hurdle in vaccine development. As ZIKV is linked to congenital malformations, one of the toughest hurdles to overcome is the need for a vaccine which is safe for pregnant women and their offspring. Another key challenge in designing a vaccine against ZIKV is the possible risk of antibody-dependent enhancement of heterologous flavivirus infection such as dengue due to the sequence and antigenic similarity between them. This has clearly been showcased in the clinical trials of a dengue vaccine developed by Sanofi [21].

## 4. Structure of Zika Virus

**Figure 1:**
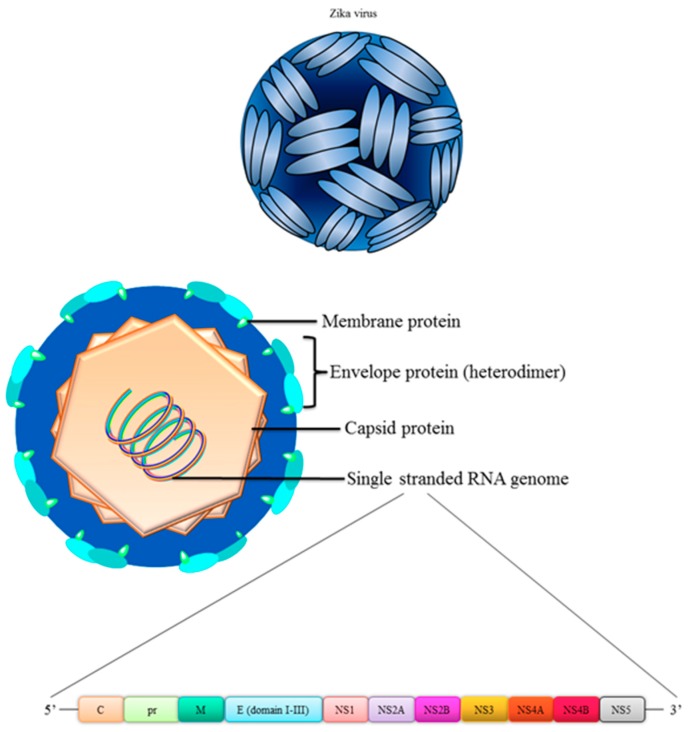
Structure of ZIKV.

A clear understanding of structure-based functional analyses of the virus is needed in order to design a vaccine against ZIKV. ZIKV has a structure akin to other flaviviruses, an icosahedral symmetry with a radius of 220 Å [22] (Figure 1). The full genome of ZIKV contains 11,000 bases which is enclosed by a lipid membrane encoding three structural proteins i.e. capsid (C), pre-membrane (prM), envelope (E) and seven non-structural (NS) proteins i.e. NS1, NS2A, NS2B, NS3, NS4A, NS4B and NS5 [23,24]. The icosahedral shape of the virus is attributed to the C protein which comprises the viral capsid whilst the outer transmembrane is decorated with E and prM/M proteins [22], which is the most commonly used target in vaccine designs which is further discussed in this review. In a resting state, the immature virus contains 60 trimeric E:M heterodimers, however, these particles lose their spiky surface following maturation process and cleavage of prM [23]. Consequently, the fusion loop is exposed, allowing the particles to fuse and become infectious in the low pH environment in the endosomes.

## 5. Immunity Induced by ZIKV Vaccines 

Vaccination is the most effective strategy for combating infectious diseases and has saved millions of lives. A successful vaccine should be able to induce efficient immune responses and generate immunological memory to provide long lasting protection against the particular pathogen [25]. In this section, we discuss a brief overview of how anti-Zika vaccines should trigger an immune response leading to memory.

Most of the vaccines against ZIKV today focus on the induction of long-lived neutralizing antibody (nAb) responses. This was based on the fact that prophylactic and curative treatments with monoclonal antibodies against Zika have been shown to protect mice or NHP against ZIKV challenges [26,27] Thus Zika vaccines should induce long-lasting memory B cells. B-cells internalize, and process viruses or vaccine molecules. These cells present viral antigen-derived peptides on their MHC II. After maximum activation and amplification, most B cells will die, and a fraction of B cells will be developed into B cell memory [25,28,29]. It has to be noted that a ZIKV vaccine will also need to induce good Zika-specific helper T cell responses, since activated T-helper recognizing MHC II/antigen complex found on B-cells promotes B cell multiplication, antibody maturation and secretion [25]. 

## 6. Vaccine Formulations

### 6.1. Live Attenuated Virus (LAV) Vaccine

Live attenuated virus vaccine is arguably one of the favored vaccination strategies due to their previous success with the yellow fever virus vaccine, YF-17D, in the 1930s. A single dose of YF-17D vaccine is able to induce high titers of nAb which confers protection on at least 95% of recipients [30,31]. This strategy has been employed with many other diseases such as polio, measles and mumps [18]. Moreover, the production of attenuated vaccine is cost effective and fairly simple in comparison to other vaccine strategies.

Kwek et al., isolated a small-plaque ZIKV variant by passaging French Polynesian ZIKV isolate in Vero cells and in C6/36 cells prior to infection on BHK-21 cells [32]. These few passages resulted in a small proportion of genetic variants, of which smaller plaques were selected. The authors picked the smallest variant of ZIKV, encoded DN-2, which had a substitution of alanine to guanine at position 948. This corresponded to a methionine to valine change in the amino acid sequence of the membrane gene. Nonetheless, DN-2 was able to infect monocyte-derived APCs, human umbilical vein endothelial cells, human embryonic stem cell-derived endothelial progenitor cells and HuH-7cells at significantly lower levels in comparison to other ZIKV strains. Subsequently, A129 mice which were inoculated with DN-2 were able to survive a 10^4^ PFU challenge without any weight loss or detectable viremia in the peripheral circulation. However, in a subsequent study, 40% of mice had low levels of DN-2 RNA in the testes 15 days post-challenge. The authors also looked at attenuated maternal-fetal transmission in A129 dams following inoculation and challenge. Fetuses of DN-2 infected dams did not show any pathological differences from the uninfected dams however, one of the fetuses did succumb to infection. Another issue concerning the use of LAV in this study is the stability of the virus. The authors noted that DN-2 is stable up to 7 passages, which questions the stability of the LAV that may have resulted in genetic variants in the following passages. 

Shan et al., manipulated the complementary deoxyribonucleic acid (cDNA) clone of the ZIKV Cambodian strain FSS13025 with various deletions to mutate the local RNA structure of the viral 3’UTR which was consequently transfected into Vero cells [33,34,35]. Immunogenicity and efficacy were then tested on AG129 mice where the authors showed that the nAb titers of mice immunized with mutated virus were comparable to those induced by the wildtype. In addition, mice inoculated with the mutated virus showed a delay in the peak viremia which was a 100-fold lower than the wild type (WT) [34]. Subsequently, the mice were challenged intraperitoneally with the Puerto Rican strain (PRVABC59 ZIKV). Interestingly, the authors found that mice immunized with mutated virus, produced four-fold higher levels of IFN-γ than those immunized with the wild type. Remarkably, none of the A129 mice which received the mutated virus showed any detectable viremia in the periphery circulation or organs following challenge. The results facilitated in narrowing down the groups of mutated viruses for their potential vaccine candidate i.e. virus with 10 nucleotide deletions (del-10). Subsequently, the authors evaluated the neurovirulence of del-10 through intracranial immunization of CD-1 pups with lower virus challenging doses. A dose as low as 10 IFU of WT virus resulted in 25% mortality whereas all the pups given del-10 survived. Finally, the authors exposed *Aedes aegypti* mosquitoes to artificial blood meals containing either WT virus or del-10. Following 7 days incubation, 56% of the engorged mosquitoes were infected with the WT virus but none with del-10 mutants [34].

In a follow up study, Shan et al. also tested the efficacy of del-10 to prevent vertical transmission in C57BL/6 female mice, following anti-IFNAR treatment [35]. At day 28 post immunization, the authors noted that all immunized mice developed high titers of nAb. Subsequently, the mice were mated and monitored for vaginal plugs. Pregnant mice were challenged with mouse adapted ZIKV African strain Dakar 41519 following anti-IFNAR antibody treatment. Mice vaccinated with a single dose of del-10, had a significant reduction in viremia and in viral loads in tissues. As expected, the levels of nAb titers were inversely correlated to the levels of infectious virus. The authors subsequently assessed if del-10 was able to prevent testicular damage in male mice. Three weeks-old A129 male mice were vaccinated with del-10 and subsequently challenged with PRVABC59. No viremia was detected in mice immunized with del-10 unlike the sham vaccinated mice. In addition, there was no reduction in total sperm or motile sperm counts in comparison to healthy uninfected mice. Results with vaccinated male mice were in total contrast with the sham vaccinated mice, which saw a significant reduction in both total and motile sperm counts. In addition, the pathology of the testes also differed whereby mice vaccinated with del-10 did not show any reduction in size or weight unlike the sham treated mice. Interestingly, these results were not reproducible in older male mice as the sham vaccinated mice did not show any weight or size reduction. The others then tested their vaccine candidate on non-human primates. Primarily, the authors evaluated the level of attenuation of their vaccine and found that only one of the four rhesus macaques showed detectable viremia. Subsequently, the authors noted that the levels of nAb titers elicited in macaques vaccinated with del-10, were comparatively lower than those inoculated with the WT ZIKV. However, both groups of macaques did not display any viremia following challenge at day 56. This was in contrast to PBS vaccinated group where high levels of viremia were detected. Neutralizing antibody titers increased between 10–100 folds in mice vaccinated with del-10 however, no significant increase was seen in the group given WT ZIKV. As del-10 was unable to confer sterilizing immunity in macaques, the authors tested another candidate, annotated as del-20 (containing a 20 nucleotide deletion) as a second LAV candidate. The same sets of experiments were performed to test the safety and immunogenicity of the vaccine candidate, del-20. Male A129 mice were immunized with del-20 and by day 6, all mice had exhibited lower virus titers in comparison to WT ZIKV inoculated mice. This was as expected as del-20 was previously shown to be less attenuated than del-10 as it was less sensitive to type I interferon inhibition. Similar to the results of del-10, when young (3-week-old) male A129 mice were challenged with PRVABC59 following immunization, the mice did not show any loss of sperm counts, zero detectable viremia in testis or any changes in testis pathology. Interestingly, when neonate CD-1 mice were subjected to 10^4^ FFU of del-20 intracranially, there was a mortality rate of 29% whereas all neonates survived when given 10^3^ FFU of del-20. *Aedes aegypti* mosquitoes were then fed to a blood meal containing WT ZIKV and del-20. 50% of mosquitoes fed with WT were infected with ZIKV whereas none showed any viral RNA when fed with del-20. Finally, the authors proceeded with testing del-20 on macaques. Two of the three macaques that were vaccinated with del-20 subcutaneously exhibited low levels of viremia in various organs. Nonetheless, these NHPs soon developed high nAb titers by day 10 following vaccination. Subsequently, the three macaques were challenged with PRVABC59 on day 56 and displayed no viremia which the authors concluded that del-20 induces sterilizing immunity. In conclusion, a live attenuated vaccine undoubtedly has its advantages of able to elicit strong immune responses with a single dose, however, there are certain drawbacks to using live attenuated vaccines as well. These include its limited use in immunocompromised or pregnant patients due to the risk of adverse effects. Hence there is still a need for other avenues in order to provide complementary options for controlling ZIKV infections.

### 6.2. Messenger RNA (mRNA) Vaccines

The use of mRNA vaccines is a relatively new trend that has gained popularity after it first made headlines in the 1990’s. As the minimal genetic construct, mRNA is containing only the elements required for expression of the specific encoded protein region. In addition, mRNA is incapable of interacting with the genome but acts only as a transient carrier of information. Other advantages for its use as vaccine platforms include its safety profile; as it is non-immunogenic, there is no risk of potential infection unlike LAV [36]. 2017 saw an influx of new mRNA vaccines coming to play in targeting the disease, however, one of the disadvantages of utilizing mRNA in an approach to vaccine design is its rapid degradation by ribonucleases [37]. This setback however, could be easily tackled with the use of delivery systems as discussed in the examples given below.

Early in 2017, Pardi et al. designed a mRNA vaccine against ZIKV utilizing the prM-E glycoproteins of ZIKV H/PF/2013 with a modified nucleoside 1-methypseudourine which abrogate innate sensing and increases mRNA translation in vivo [38]. mRNA was subsequently packaged into lipid nanoparticles composed of ionizable cationic lipids, phosphatidylcholine, cholesterol and polyethylene glycol. The authors initially tested the efficacy of their vaccine candidate on C57BL/6 mice which were given ZIKV-prM-E mRNA LNP intradermally. By week 8, vaccinated mice were able to elicit high titers of IgG and nAb. Subsequently, similar studies were repeated in BALB/c mice whereby mice showed peak antibody production by week 8. The authors proceeded with a challenge study on the immunized BALB/c mice. Mice were challenged with PRVABC59 on either week 2 or week 20 following immunization. Mice immunized with mRNA LNP did not exhibit any detectable viremia in the blood whereas 8 out of 9 showed presence of viral RNA in the sham group. Consequently, the authors assessed the efficacy of their vaccine candidate on rhesus macaques. Animals were immunized i.d. with varying doses of the vaccine. Interestingly, there were no significant differences in E-protein specific IgG and nAb titers induced by the varying amounts of vaccine given to the various groups of macaques. A challenge with the PRVABC59 virus was conducted 5 weeks following immunization. Negative control animals which did not receive the vaccine were susceptible to infection by day 3 post infection. In contrast, all vaccinated animals, regardless of the amount of vaccine received, were protected against ZIKV infection with one exception. A macaque that received the highest dose of vaccine had low transient viral RNA detected on day 3 however, it resolved by day 5. Unsurprisingly, this macaque exhibited one of the lowest nAb titers amongst the group of vaccinated animals. Further studies need to be conducted with bigger sample size as a single blip in this study, had implications in determining correlates of protection.

In 2017, Richner et al., engineered a lipid nanoparticle encapsulating modified mRNA encoding ZIKV prM/M and E genes from the Asian strain together with the signal sequence of human IgE (IgE_sig_-prM-E LNP) [39]. The vaccine candidate, IgE_sig_-prM-E LNP, was initially assessed for its efficacy in AG129 mice. Mice were injected intramuscularly (i.m.) with either a single or double dose of the vaccine. The groups receiving two doses were able to elicit high titers of sera nAb. Subsequently, these mice were challenged with ZIKV P6-740 (Malaysian strain) on day 42. Unsurprisingly, mice which received two doses of the vaccine all survived with the exception of a single animal. Subsequently, the authors tested the vaccine on immunocompetent C57BL/6 mice. Eight week old male mice were given two doses of the vaccine prior to receiving anti-ifnar1 antibody and challenged. All mice survived without detection of viremia post infection. As the fusion loop (FL) in DII of the E protein is immunodominant in humans, there is a possible risk that vaccination with ZIKV full protein could elicit cross-reactive antibodies which may enhance other flavivirus infection through ADE. Hence, the authors generated mutations in the mRNA vaccine (IgE_sig_-prM-E FL LNP) and a series of Japanese encephalitis virus (JEV) leader sequence replacing the IgE in mRNA LNPs i.e. JEV_sig_-prM-E-FL LNP, to eliminate the antibody reactivity of FL-specific antibody. BALB/c mice vaccinated with JEV_sig_-prM-E LNP showed the highest level of protection (one mouse had viral RNA detected in the brain) following challenge with ZIKV Dakar 41,519 after anti-Ifnar1 treatment. Unfortunately, vaccine candidates with FL mutations were unable to confer full protection as viral RNA was detected in some animals albeit at much lower levels in comparison to the placebo LNPs. Similar to other studies, the authors found that levels of nAb were inversely correlated to the levels of ZIKV RNA in the sera and tissues. In order to evaluate whether the mutated vaccines were able to induce cross-reactive antibodies, sera of vaccinated mice were incubated with dengue virus serotype 1 (DENV-1) RVP prior overlaying on Fcγ receptor IIA-expressing K562 cells. As expected, sera from mice immunized with the FL mutation displayed a lower peak sera enhancement titer (PET) i.e., a reduction in cross-reactivity. Subsequently, authors passively transferred sera from vaccinated mice to AG129 mice 1 day prior to challenge with DENV-2. Unsurprisingly, at least 80% of mice that had sera from the FL mutations survived with lower morbidity clinical scores in comparison to those mice given the sera from full IgE_sig_-prM-E LNP.

### 6.3. DNA Vaccines

DNA vaccines are of the earliest vaccine platforms to be proposed for human clinical trials following the ZIKV outbreak (clinicaltrials.gov). The use of genetically engineered DNA plasmids encoding various antigens to induce both humoral and cellular responses has been explored against various infectious diseases caused by parasites [40,41], bacteria [17,42] and other viruses [43,44]. Here, we explore the recent advances of DNA vaccines against ZIKV infection focusing on those that have reached studies on NHPs and human clinical trials.

Larocca et al. designed their DNA vaccine based on the pre-membrane and envelope (prM-Env) of the Brazil strain BeH815744, scrapping the first 93 amino acids of prM [45]. The authors also tested a few other vaccine candidates, carrying various mutations in the plasmid DNA prM-Env i.e. lacking prM and/or lacking the transmembrane region or the full stem of Env, in parallel with their initial candidate. The immunogenicity of the various DNA plasmids was assessed using BALB/c mice. Mice given prM-Env vaccine intravascularly was able to elicit higher Env-specific IgG titers in comparison to all other groups. In addition, the prM-Env vaccine was also able to induce ZIKV-specific nAb and Env-specific CD8^+^ and CD4^+^ T lymphocyte responses. Subsequently, a challenge study was performed to determine the protective efficacy of DNA vaccine against a challenge using ZIKV PRVABC59 in addition to Brazil/ZKV2015. Mice that were vaccinated with the complete prM-Env were completely protected following challenge from the two different strains of ZIKV whereas the mice vaccinated with mutated/truncated versions of the DNA vaccine, did not show complete protection against ZIKV-BR. Furthermore, the authors tested the protective efficacy of the DNA vaccine on SJL and C57BL/6 mice which resulted in the same outcome whereby all mice given prM-Env DNA vaccine were fully protected. Subsequently, Larocca and colleagues passively transferred purified IgG sera from BALB/c vaccinated with prM-Env mice to naïve mice. Mice receiving sera Env-specific antibody titers of above log 2.35, were protected against ZIKV-BR challenge. Next, prM-Env vaccinated mice were depleted of CD8^+^ and CD4^+^ T lymphocytes prior challenge. Interestingly, depletion of T lymphocytes did not abrogate the protective efficacy of the prM-Env DNA vaccine upon challenge. There was no detectable viremia following challenge with ZIKV-BR however; viral RNA could be detected in the unvaccinated group.

Abbink et al., extended this study by testing the protective efficacy of prM-Env DNA vaccine in rhesus macaques [46]. Monkeys received two doses of the DNA vaccines intramuscularly four weeks apart. Surprisingly, minimal nAb titers were only present on week 4 following primary immunization, however titers increased following the boost. Despite low levels of nAb detected in the animals, the monkeys had a 100% protection following a subcutaneous challenge with 10^3^ PFU ZIKV-BR.

Muthumani et al. reported on a similar vaccine construct targeting the pre-membrane and envelope proteins (prM-E) of ZIKV [47]. However, they added an IgE leader sequence to facilitate better expression similar to the concept used by Richner et al. [39] for their mRNA vaccine. The authors first tested the ability of the DNA vaccine to induce antigen specific T cells following intramuscular vaccination. Splenocytes were harvested and tested for their ability to secrete IFN-γ following ex vivo exposure to various peptide pools. Further evaluation of vaccine immunogenicity found that the vaccination increased the proportion of vaccine-specific T cells which expresses TNF-α and IFN-γ. Subsequently, the authors tested their vaccine efficacy by administering C57BL/6 mice with DNA vaccine intramuscularly in a prime boost regimen. ZIKV-E-specific IgG antibody titers were highest following a boosting, moreover titers were maintained even past day 60 post immunization. Subsequently, the vaccine efficacy was tested on IFNAR-deficient mice. Similarly, sera from vaccinated animals had detectable anti-ZIKV IgG by day 14 and also able to neutralize ZIKV though at relatively lower levels in comparison to C57BL/6 mice. Mice were subsequently challenged with PR209 (Puerto Rico) Zika virus following 2 doses of immunization. By the end of 3 weeks post challenge, all mice that were received the vaccine survived compared to mice control mice which had a 30% survival rate. Furthermore, the vaccinated animals did not show signs of morbidity unlike mice in the control where all animals were presented with either weight loss or struggling with mobility. Subsequently, the authors reduced the dose regimen by subjecting IFNAR-deficient mice to a single immunization prior challenge. Even though mice vaccinated with the DNA vaccine survived, viral RNA could be found in 40% of the mice. Muthumani et al. then tested their vaccine on NHPs. Macaques were immunized using intradermal electroporation with the DNA vaccine twice, two weeks apart. Sera and peripheral blood mononuclear cells (PBMCs) were collected on week 6 following immunization. PBMCs stimulated with prM-E peptide pools showed that ZIKV prM-E immunization was able to induce robust anti-ZIKV T cell responses in vaccinated RM. In addition, all vaccinated animals also had significant increase in nAb titers. The immune sera of macaques were able to prevent ZIKV infection in Vero, neuroblastoma and neural progenitor cells in vitro when tested via IFA. Sera from vaccinated Rhesus macaques were adoptively transferred into IFNAR-deficient mice a day prior to challenge. Mice which received control sera or PBS were all dead by day 8, however 80% of the mice receiving sera from vaccinated macaques survived. The study was further carried by a clinical trial (NCT02809443) [48], whereby the vaccine was administered intradermally in a prime-boost regimen consisting of 3 doses of vaccine. None of the patients reported any systemic adverse effect following vaccination. At 14 weeks post vaccination, all participants had measurable ZIKV E protein specific antibody titers; however, not all of the vaccinated individuals had nAb. Nonetheless, sera from the participants were passively transferred to IFNAR-deficient mice prior to challenge with a lethal dose of clinical strain ZIKV PR209 (Puerto Rico). Mice that received baseline sera or PBS, died within 9 days of infection. Whereas 92% of the mice receiving sera from vaccinated individuals survived past day 14. Interestingly, mice which survived included those which received sera from individuals with no detectable nAb. This provides further question for the use of nAb, determined in vitro, as a correlate of protection.

Another DNA vaccine study conducted by Dowd et al. utilized the prM-E sequences inserted into the cytomegalovirus immediate promoter cloned into plasmid VRC8400 [49]. However, the difference between the constructs as published by Larocca et al. [45] and Abbink et al. [46], Dowd et al. included the whole prM sequence which was based on the French Polynesian isolate H/PF/2013 virus and also replaced the signal sequence of ZIKV prM with analogous region of JEV annotated as VRC5283. Based on VRC5283, Dowd et al. designed a second vaccine construct which replaced the final 98 amino acids of the stem and transmembrane regions of E protein with corresponding JEV sequence (VRC5288). Both vaccines were able to induce of ZIKV-specific nAb production following a single immunization. Subsequently, the authors optimized the dosage and regimen of their vaccine candidates. Macaques were administered either two doses of 1 mg VRC5283 or 4 mg of either VRC5283 or VRC5288. Another group of monkeys received a single 1 mg dose of VRC5288 and control animals were immunized with the empty vector. Animals which were vaccinated with either VRC5283 or VRC5288 had significantly higher nAb titers compared to the control. Moreover, macaques that received two doses of either VRC5283 or VRC5288 had nAb titers which were significantly higher than those that received a single dose. All animals were challenged 8 weeks following the first dose of vaccination with PRVABC59 ZIKV. Only animals which received double doses of vaccine were protected with the exception of one animal which had blips on day 3 and 7. All other animals i.e. those receiving just one vaccination and the control group, showed viremia by day 3. The vaccine constructs VRC5283 and VRC5288 advanced to phase 1 clinical trials (NCT02840487 and NCT02996461) [50]. None of the healthy volunteers whom were given either a two dose or three dose regimen reported any adverse side effects. Volunteers were either given the vaccine intramuscularly using a needle syringe or a needle free device (Stratis device, Pharmajet). Sera nAb titers could be detected 4 weeks following immunization. However, the only group where all volunteers showed detectable levels of nAb were those given three doses of VRC5283 using the needle-free device. This would have matched the NHP challenge model where the macaques which were administered with VRC5283 showed better protection compared to VRC5288. However, the authors did not utilize the needle free device when administering VRC5288 to any of the patient groups, which makes it difficult to compare the vaccine efficacy of the two different candidates in humans.

### 6.4. Adenovirus Vector Based

Adenovirus vectors have been well studied as a vector for gene and cancer therapy and also vaccines whereby the vector expresses an unknown antigenic protein. Apart from its extensive safety profile, the advantages of utilizing adenovirus vectors are that it is relatively stable, easy to attain high titers and able to infect multiple cell lines which attributes to its potency.

Xu et al. manipulated a recombinant chimpanzee adenovirus type 7 (AdC7) by cloning the gene encoding the signal peptide of JEV and full length ZIKV/M/E glycoproteins (AdC7-M/E) [51]. Vaccine immunogenicity was assessed in two groups of BALB/c mice immunized intramuscularly with either a high or low dose adenovirus particles of recombinant AdC7-M/E. A booster dose was performed 4 weeks following primary immunization. As E-specific antibody titers and nAb titers following primary immunization with high dose of virus particles were considerably high titer. There were no significant difference titers following a booster dose. Subsequently, sera from vaccinated BALB/c mice were tested for its ability to cross neutralize heterologous ZIKV strains, MR766, FSS13025 (Cambodia strain) and SMGC (Asian strain). The authors then tested the efficacy of the vaccine in mice lacking type 1 interferon. IFNAR-deficient mice were given a single dose of AdC7-M/E and antibody titers measured were of similar values as the BALB/c mice. Protection efficacy of the vaccine was evaluated by challenging intraperitoneally with ZIKV-SMGC IFNAR-deficient mice that have been immunized with one dose of vaccines. All sham mice succumbed to infection and died by day 8, however all vaccinated mice (low and high dose) survived with no weight loss. The authors then re-challenged the surviving vaccinated mice with a lethal dose of ZIKV. Astonishingly, there were no viral RNA detected in either the blood or organs (brain, spleen, spinal cord, testes, liver) of immunized mice. In contrast, sham immunized mice showed high levels of viral RNA in the blood and tissues, especially the testes whereby there was fibrosis of the tissues and presence of inflammatory cells. A passive immunization study was conducted to evaluate the protective efficacy of the vaccine. Sera from BALB/c immunized mice were passively transferred intraperitoneally to immunodeficient mice and subsequently challenged later with a lethal dose of ZIKV. All mice receiving sera from vaccinated animals survived, however a slight decrease in weight could be seen between days 5 and 8 post immunization but quickly rebounded and increase steadily after day 8. Sham-immunized mice succumbed to infection and died within 10 days.

In an independent study conducted by Guo et al. the authors manipulated a more commonly used adenovirus, human type 5, expressing the pre-membrane and envelope proteins of ZIKV strain MRS_OPY_Martinique_PaRi_2015 [52]. Three different constructs were designed, with codon optimization to enhance transgene expression; Ad5-Luciferase (control), Ad5-Env and Ad5-Sig-prM-Env. The authors first evaluated the vaccine efficacy by giving mice a single dose of either vaccine or control (Ad5-Luc) intramuscularly. Vaccination with both vaccine constructs were able to induce anti ZIKV-Env-specific antibodies which were significantly higher than Ad5-Luc. Similarly, when the authors phenotyped T cells by flow cytometry and performed ELISPOT assay to evaluate the cellular immune responses elicited by the vaccines, the levels of IFN-γ-, TNF-α-, IL-2-, and CD107a-positive CD8^+^ T cells and IFN-γ- and TNF-α-positive CD4^+^ T cells in the vaccinated mice were significantly higher than the control group. Nonetheless, for both immune responses, there were no significant differences in antibody production or cytokine stimulation between the two vaccinated groups. Unlike most studies whereby authors use immunodeficient mice, Guo et al. subjected mice to corticosteroid (dexamethasone) therapy to mimic the scenario of patients with immunosuppression. The two vaccines were able to elicit similarly high levels of anti-ZIKV-Env-specific binding antibodies in sera at 2 weeks post vaccination even under glucocorticoid treatment. Interestingly, mice given Ad5-Sig-prM-Env had significantly higher titers of anti-ZIKV specific nAb which also explain the difference of viral RNA levels between the two vaccinated groups i.e. no detectable viral RNA in sera and tissues of Ad5-Sig-prM-Env vaccinated mice and low levels in the Ad5-Env vaccinated mice following challenge with PRVABC59. A similar result was seen when A129 mice were vaccinated and challenged. Interestingly though, both vaccinated groups including the negative control, showed weight loss, however, only mice vaccinated with Ad5-Sig-prM-Env had no detectable viremia in sera or tissues. In addition, all mice vaccinated with Ad5-Sig-prM-Env survived lethal challenge whereas only 83% of those vaccinated with Ad5-Env survived which could once again be due to higher levels of sera anti-ZIKV specific nAb in Ad5-Sig-prM-Env vaccinated mice. The authors subsequently passively transferred sera of vaccinated mice into naïve A129 mice prior to lethal challenge. Mice receiving sera from Ad5-Sig-prM-Env vaccinated mice had a 100% survival rate with minimal weight loss and zero detectable viral RNA in blood or tissues 6 days post infection. In contrast, mice receiving Ad5-Env only had 66% survival rate, with detectable viral RNA in blood and tissues and weight loss. Nonetheless, levels of detectable viral RNA were still significantly lower than those given sera of sham mice. This study was the only study that tried to mimic vaccination efficacy in immunosuppressed patients which is critical prior to testing the vaccine in phase 2 clinical trials. There were differences in protective efficacy of the two vaccine constructs, adenoviruses expressing prM-EnV and the -Env region, which suggests that the prM region is fairly important which may be resulted from the particulate form antigen (prM-EnV) and the non-particulate antigen (-Env). However, though there were no detectable viremia or viral RNA in the tissues, the mice did suffer weight loss following challenge, which suggests that the animals did succumb to infection but were able to recover.

### 6.5. Subunit Vaccines

Subunit vaccines comprise of a fragment of a pathogen, i.e. protein, or peptides. Unlike live attenuated vaccines, subunit vaccines are generally a safer choice, however they tend to be less immunogenic as well. Hence an adjuvant and/or multiple doses are required.

To et al. utilized a recombinant protein derived from the envelope protein of ZIKV (ZIKV E) based on the French-Polynesian strain [53]. Immunogenicity was tested in various mouse models including Swiss Webster (Swiss), BALB/c and also C57BL/6J mice. The authors initially tested their vaccine candidate, a ZIKV E-based recombinant protein, either alone or formulated with various adjuvants including alum-based adjuvants (Alhydrogel or Imject) or CoVaccine HT in Swiss mice. Mice that were immunized with CoVaccine HT elicited the highest ZIKV E IgG antibody which was significantly higher than those immunized with the ZIKV E together with alum adjuvants. Subsequently, when the authors tested their various vaccine candidates in BALB/c and C57BL/6 mice, both formulations i.e. alum and CoVaccine HT, of ZIKV E protein were able to induce high antibody titers with them showing no significant difference between the two formulations. Neutralizing antibodies were measured using a plaque reduction neutralization (PRNT) test following two doses of immunization of which the authors found comparable titers of nAb in both groups. Subsequently, protection studies were performed using BALB/c mice. Mice were intravenously challenged with PRVABC59 virus, four weeks post immunization. Groups that were given two doses of vaccines were fully protected unlike those given a single dose. Subsequently, authors tested if antisera from immunized mice would confer passive protection against ZIKV challenge. 10 µg of pooled sera, together with the various adjuvants, was administered intraperitoneally into BALB/c mice a day prior to challenge with ZIKV. The authors concluded there was a decrease in ZIKV E specific IgG antibody titers 14 days post infection in the group of mice receiving the antisera. Seroconversion was used as a gauge of virus infection in unprotected mice, therefore the authors looked at antibody production against ZIKV NS1. All animals presented with antibodies against ZIKV NS1 which suggested that mice receiving sera antibodies were unprotected.

Another subunit vaccine derived from the ZIKV E protein of strain PRVABC59, was studied by Yang et al. [54]. Unlike the previous study, Yang et al. utilized a specific portion of the E protein i.e. the Domain III (DIII). ZIKV E DIII was produced in *E. coli* expression systems and subcutaneously administered into C57BL/6 mice together with adjuvant (alum or TitreMax, a water in oil adjuvant). Mice were given different doses of DIII. There was no significant difference in sera antibody titers between groups of mice given different doses of antigen following the third immunization. The groups of mice immunized with adjuvants were able to induce high titers of both IgG_1_ and IgG_2c_ antibodies with a bias towards IgG_1_ suggesting a Th-2 type response. Splenocytes from mice immunized with the vaccine candidates were able to induce IFN-γ, IL-6 and IL-4 production in contrast to PBS immunized mice, when stimulated with either EDIII or non-specifically with Con A. For both vaccine candidates, more than 80% of the virus was neutralized in vitro. Subsequently, the authors tested the possibility of antibody dependent enhancement activity of IgGs by incubating sera of immunized mice with DENV-2 E domain protein. The mixture was subsequently incubated with K562 cells displaying the human FcγR. The authors concluded that the purified IgGs from sera of mice immunized with ZIKV D III vaccine did not display any ADE activity which is comparable to the negative control. However further studies in in vivo models are needed to prove the vaccine is able to confer protection.

Tai et al. conducted a long-term vaccination study using various fragments of recombinant ZIKV EDIII (ZikaSPH2015 strain) which were codon optimized as vaccine candidates [55]. To test the efficacy of their vaccine candidates, BALB/c mice were given four doses of the recombinant protein together with alum or monophosphoryl lipid A (MPLA). Sera from immunized mice were collected and IgG antibody titers were determined. All of the vaccine constructs tested were able to induce nAb following immunization. However, nAb titers following immunization of recombinant protein E298-409 were significantly higher than the other two; E296-406 and E301-404. Subsequently, long term antibody titers were evaluated. Sera obtained at 10 months post immunization showed that all vaccine constructs were able to elicit long term EDIII however, constructs E298-409 and E301-404 were able to significantly induce higher E-specific IgG titers in comparison to E296-406. The authors then tested if sera from immunized mice were able to protect pups against ZIKV infection. Pups born to BALB/c mice 7 months post immunization, were challenged with two human epidemic strains FLR (Colombia) and R103451 (Honduras). All pups which were born to mice immunized with ZIKV EDIII fragments survived except for those which were born to those immunized with E301-404 which only saw 83% survival when challenged with the Honduras strain. In contrast, pups from PBS immunized mice died. Next, the authors conducted an adoptive transfer study using sera from mice immunized with E298-409, which gave the highest titers of nAb. Seven-day old BALB/c pups were given immune sera and challenged with ZIKV FLR and R103451. Unfortunately, only 80% of pups survived in both challenged groups. Consequently, the authors tested the protective efficacy of sera obtained from adult immunized mice transferred to A129 mice. Anti-E298-409 sera equal to 10^5^ ZIKV EDIII-specific IgG titer was transferred to A129 mice. A 100% survival was seen when the challenged with both ZIKV strains. However, viral RNA was present in the brain, lung, liver, spleen and kidney 5 days post infection in addition to tissue damage. The overall study identified that E298-409 as a promising vaccine candidate which is able to stimulate high titers of maternal nAb and confer protection to newborns. However, it is not known if maternal Ab are able to confer protection to the fetus if the pregnant female mice were subsequently challenged with ZIKV. This would have been an important study as we know that ZIKV is able to cross the placenta and cause fetal abnormality.

### 6.6. Combinatorial Vaccines

While the development of single ZIKV vaccine is ongoing, a multiple antigenic approach following the measles mumps, rubella and varicella (MMRV) vaccine was being explored by Chattopadhyay and colleagues [56]. In their combinatorial vaccine against Chikungunya virus (CHIKV) and ZIKV, they utilized recombinant vesicular stomatitis virus (VSV) expressing CHIKV envelope polyprotein and ZIKV E protein. Sera from BALB/C mice immunized with a single dose of 10^7^ PFU of recombinant VSV vaccine were able to neutralize 70% of ZIKV (Brazilian strain PE243). Whereas those which were given two doses of the vaccine were able to neutralize 80% of ZIKV. In addition, sera from the single immunization of BALB/C were also able to neutralize 100% of VSVΔG-eGFP/CHIKV pseudotype. In order to determine the protective efficacy of the vaccine, 7 week old A129 mice were immunized with 1 intramuscularly prior to challenge with either MR 766 Zika virus or CHIKV. Interestingly, none of the vaccinated mice showed signs of viremia following infection with either viruses. Although the authors proved that the mice were free from infection, the mice were past 15 weeks old when challenged. In this particular murine model, it is known that ZIKV infection would not have resulted in death of mice, but they would only show transient signs of illness following infection. Nonetheless, the negative control mice given CHIKV succumbed to infection by day 3 whereas all immunized mice survived. Overall, the study managed to prove that the vaccine was able to prevent viremia in immunocompromised mice, though the authors did not mention of any physical signs of infection. However, there was no proof that the vaccine would be useful in inducing production of maternal nAb in pregnant dams which is able to confer protection to newborns. In addition, it would be interesting to also study the possible deviations on the vaccine effects following co-infection with CHIKV and ZIKV.

## 7. The Pros and Cons of Various Vaccine Platforms

The most common vaccine platform used today is the live attenuated vaccine. This includes the chickenpox, rotavirus and the MMRV vaccines. The advantage of utilizing live attenuated vaccines includes its strong humoral and cellular mediated response triggered. Hence, typically a single dose of vaccine is needed. This proves useful in developing countries where the traveling time and cost of returning to the clinic may be a problem. Nonetheless, the platform is not without its major flaw where the organism may revert and cause more harm or immunosuppression [57]. This is a crucial point to consider in the context of a ZIKV vaccine as the more susceptible group to vaccinate would be the expectant mothers who already have a weakened immune system. Furthermore, it is known that ZIKV persists for months in the semen and urine [58], which undoubtedly pose a huge risk of LAV shedding into the semen.

Unlike live attenuated vaccines, nucleic acid vaccines are non-immunogenic on their own yet they are also able to induce strong humoral and cellular mediated responses [59]. The major drawback of ribonucleic acid vaccines is its ability to avoid degradation by circulating ribonucleases. In order to deliver such vaccines, a delivery system is required. One major cause of concern is the possibility of an insertion of foreign genomic sequences into the host genome which may alter the resulting immune responses [60]. Even so, this is one of the more common vaccine platforms being used in the search for a ZIKV vaccine due to its safety profile.

Recombinant subunit vaccines include those manipulating the viral/bacterial vector in order to express the protein(s) of interest. The advantage of utilizing recombinant vaccine lies in its safety profile and low production cost. However, due to its weak immunogenicity, there is a need for these vaccines to be delivered with a potent adjuvant to boost the immune responses triggered by the antigen [61]. Conversely, with recent advances, we now have live recombinant vectors e.g. adenoviral vectors, which assist in improving the immunogenicity of the vaccine. The recombinant adenoviral vectors are widely used today thanks to its high transduction efficiency and transgene expression. However, as most of the population have been exposed to adenovirus, there is a likelihood for pre-existing immunity against the vector. This has been proven detrimental in a human immunodeficiency virus (HIV-1) phase IIb vaccine trial whereby the vector-based vaccines provided favorable conditions for HIV-1 replication [62]. The risk of utilizing such recombinant viral vector vaccine may not be of concern for immunocompetent individuals, however, as the vaccine is more important to expectant mothers, the risk may outweigh the benefit.

Unfortunately, we are unable to provide a detailed summary of every vaccine study that has been published, however, relevant references are provided in Table 1. Nonetheless, each individual vaccine platform has its own pros and cons which may not be suitable for the entire population. However, as the essential targets for the vaccine are pregnant mothers, the need to ensure the safety profile is crucial to not harm the mother and fetus in addition to the vaccine being able to induce nAb production and prevent vertical transmission.

## 8. Conclusions

The quest for a vaccine against ZIKV has progressed tremendously since the outbreak of 2014. However, one of the biggest conundrums in vaccine research is finding a vaccine that is applicable to the vast majority. Until today, most of the vaccine study focuses on the protective efficacy without considering the need to vaccinate immunosuppressed patients as well. As Zika research progresses, it is evident that the two different lineages found throughout the world have various infectivity and potency which needs to be considered when performing challenge experiments. The current trends of vaccines against ZIKV mainly concern the pre-membrane, membrane and envelope proteins as the target epitopes for vaccine candidates due to the natural structure of the virus. It is essential to note that most of these studies focus on a single vaccine candidate, with the more comprehensive approach performed by Abbink et al. which compared three different vaccine approaches i.e. purified inactivated vaccine, DNA vaccine and adenovirus vector vaccine [44] in a single study. Although many dwell on neutralizing antibodies as a predictor for protective efficacy, correlates of protection remain to be defined. As a number of vaccines are already being tested or completed the first phase in clinical trials, it is likely that a vaccine will be available in the coming future.

## Figures and Tables

**Table 1:** Types of vaccines and phases of research.

Vaccine	Type of Vaccine	Sponsor	Phase
ZIKA-001 *	DNA plasmid vaccine	GeneOne Life Science	Phase 1
GLS-5700 * [48]	DNA plasmid vaccine	GeneOne Life Science	Phase 1
mRNA-1325*	mRNA vaccine	Moderna Therapeutics	Phase 1/2
MV-ZIKA *	Recombinant measles-Zika vaccine	Themis Bioscience	Phase 1
VRC-ZKADNA090-00-VP * [50]	DNA vaccine	National Institutes of Allergy and Infectious Diseases (NIAID)	Phase 1/2
ZPIV *	ZIKV purified inactivated vaccine	NIAID	Phase 1
PIZV *	ZIKV purified inactivated vaccine	Takeda	Phase 1
VLA1601 *	ZIKV purified inactivated vaccine+alum	Valneva Austria GmbH	Phase 1
VRC-ZKADNA085-00-VP * [50]	DNA vaccine	NIAID	Phase 1
rZIKV/D4Δ30-713 *	Live attenuated ZIKV vaccine	NIAID	Phase 1
**Type of vaccine**		**Target**	**Phase**
DNA Vaccine		Pre-membrane/Membrane (PrM) Envelope (E) protein [45,46,47,48,49,50,63,64]	Pre-clinical mouse model NHP
Live attenuated Zika vaccine		Whole virus [32,33,34,35,65,66,67]	Pre-clinical mouse model NHP
Live ZIKV chimeric vaccine (YF 17-D attenuated backbone)		Whole virus [68,69]	Pre-clinical mouse model
Adenoviral vector type 26		Whole virus [70]	Pre-clinical mouse model NHP
Recombinant protein		E protein [54,55,71,72,73] Capsid (C) PrME + NS2B/NS3 proteins [74]	Pre-clinical mouse model
Adenoviral vector type 2		PrME protein + NS1 protein [75]	Pre-clinical mouse model
Recombinant VSV		Membrane (M) E [56] PrME [76] PrME + NS1 [77]	Pre-clinical mouse model
Chimpanzee adenoviral vector		PrME protein [78]	Pre-clinical mouse model
Outer membrane vesicles from Neisseria Meningitidis (Proteosome)		Whole virus [79]	Pre-clinical mouse model
“Sementis Copenhagen Vector” (SCV) system		PrME protein [80]	Pre-clinical mouse model
Human adenoviral vector type 5		PrME protein [52]	Pre-clinical mouse model
Recombinant chimeric ZIKV vaccine candidate (JEV backbone)		PrME protein [81]	Pre-clinical mouse model
Recombinant chimpanzee adenovirus type 7		PrME protein [51]	Pre-clinical mouse model
Modified Vaccinia Ankara (MVA) vector		NS1 protein [82]	Pre-clinical mouse model
Plant produced ZIKV E protein		E protein [83]	Pre-clinical mouse model
Virus like particles		PrME protein [84]	Pre-clinical mouse model
RNA nanoparticle vaccine		PrME protein [85]	Pre-clinical mouse model
Insect cell-produced subunit vaccine		E protein [53,86]	Pre-clinical mouse model

* Adapted from clinicaltrials.gov as of 18 October 2018.
